# Household assets, expenditures, and longitudinal memory changes among older adults in rural South Africa

**DOI:** 10.1093/geroni/igaf122.2035

**Published:** 2025-12-31

**Authors:** Chuwen Zhong, Xuexin Yu, Lindsay Kobayashi

**Affiliations:** University of Michigan, Ann Arbor, Michigan, United States; Columbia University Mailman School of Public Health, New York, New York, United States; University of Michigan, Ann Arbor, Michigan, United States

## Abstract

The relationships between cognitively-stimulating types of household assets and expenditures with cognitive function and decline among older adults in low-income settings are unclear. We investigated how household assets and expenditures relate to cognitive function and decline over time among 3,942 participants aged ≥40 years in the population-representative HAALSI cohort study in rural Agincourt, South Africa (three study time points over 8 years from 2014/15 to 2022). Household assets were categorized as the ownership of each of a radio, cellphone, smartphone, refrigerator, vehicle(s), and stove (yes/no for each) and monthly expenditures were on each of food, communication, transportation, and entertainment (measured continuously in units of 1000 RAND). Latent memory z-scores were derived from immediate and delayed recall trials at each time point. Multivariable-adjusted linear mixed-effects regression models were applied to examine associations. Mean baseline age was 63 years (SD: 12.5 years); 53% were female (2,098/3,942). Ownership of a refrigerator, a smartphone, or a vehicle, and higher expenditures on food, communication, and transportation were each associated with higher baseline memory scores. Owning a refrigerator, a vehicle, a radio, and having greater expenditures on food, communication, and transportation were each associated with a slower rate of cognitive decline over time. Consistent with cognitive reserve theory, findings indicate that improvements in household assets and expenditures, especially related to food storage, communication, and transportation, may help to delay cognitive decline in this low-income setting.

